# The frequency of injuries of Iranian male professional soccer players: a cross-sectional study

**Published:** 2024-07

**Authors:** Tohid Seif Barghi, Salman Khazaei, Bijan Heydari, Homa Naderifar

**Affiliations:** ^*a*^ Sports Medicine Research Center, Neuroscience Institute, Tehran University of Medical Sciences, Tehran, Iran.; ^*b*^ Department of Epidemiology, School of Public and Social Determinants of Health Research Center, Hamadan University of Medical Sciences, Hamadan, Iran.; ^*c*^ Department of Orthopedics, School of Medicine, Besat Hospital, Hamadan, Iran.; ^*d*^ Hearing Disorders Research Center, Hamadan University of Medical Sciences, Hamadan, Iran.

**Keywords:** Injuries, Sports, Soccer

## Abstract

**Background:**

Soccer is known to be a sport that carries a high risk of injuries due to its physical nature, involving intense contact and rapid movements like acceleration, deceleration, jumping, and sudden changes in direction. Compared to other sports, soccer is considered a contact sport with a height-ened injury risk. The primary objective of this study was to assess the prevalence of sports injuries among professional soccer players.

**Methods:**

In this cross-sectional study, 56 male professional footballers participated to assess their injuries. The Pre-Competition Medical Assessment (PCMA) was utilized as the standard protocol for evalu-ating the athletes. The study examined variables such as injury type, player position on the field, and body mass index (BMI) in accordance with the FIFA-recommended PCMA guidelines. Student t-test was used to compare demographic characteristics injured and healthy athlete. Chi square and fisher exact tests were used to assess the association between player position, injury type and BMI classification.

**Results:**

In this study, the mean age of the soccer players was 19.38 ± 1.30 years, with a history of par-ticipating in 3 to 60 matches. The highest incidence of injuries was recorded in the knee (48.21%) and ankle (30.36%), respectively. A significant relationship was found between the players' positions on the field and knee pain, with strikers experiencing more knee pain compared to players in other positions (p less than 0.04). However, no significant association was observed between the players' BMI and the occurrence of sports injuries (p greater than 0.05).

**Conclusions:**

Given the widespread occurrence of knee injuries among footballers, particularly in attacking players, proper planning and appropriate training protocols would be useful to prevent knee injuries and reduce treatment costs.

## Introduction

Soccer is a popular sport closely associated with fast movements and runs, such as acceleration, deceleration, jumping, and sudden, and quick direction changes. It is categorized as a contact and high-risk sport in which there is a high prevalence of sports injuries.^1^ Studies have indicated that 61-95 percent of soccer players have experienced at least one kind of sports injury during a season.^2,3^ Moreover, previous studies reported that the prevalence of soccer injuries was in the range of 17-24 injuries per 1000 match hours,^4,5^ each male player is confronted with an injury annually , and the number of injuries in competitions is four to six times as much as training time.^6,7^ Owing to the high rate of injuries, a team undergoes high medical costs for the recovery of the injured player.^8^ According to Karanian et al.^9^ in the Pro League Soccer of Iran, the cost of treating the players’ injuries is prohibitive. They further stated that the prevalence of knee injury, and consequently its treatment were more costly than other injuries.^10^ In addition, in some cases, an injured player may have to rest for a long period (more than a month) to recuperate, which is not cost-effective in today's soccer.^11^

Sprains of the ankle, knee, hamstring, and groin muscles are four common injuries that encompass more than 50% of all soccer injuries.^4^ Approximately 50% of injuries in soccer occur following direct contact between players and occur during processes, such as collisions and tackles, while in non-collision injuries transpire in situations, such as running, shooting, spinning, and heading.^12,13^ Several studies have investigated the sports injury rate among professional soccer players around the world, the majority of which have announced lower extremities as the most common area for the incidence of injuries.^4,14^ For example, Angorani et al. noticed the most common injuries in the lower body as well as the knee of 400 Iranian Pro League Soccer players.^15^ Rahnama et al.^16^ and Maghami et al.^17^ obtained the same results. Some other studies have reported leg (19.6%)^18^ and ankle^16^ as the most injured body parts. 

Although studies support the hypothesis that soccer players’ position affects their injury rate, there is disagreement as to which position is the most susceptible to injury.^19-22^ Some studies showed that midfielders could be the most susceptible to the extent of 42%,^15^ 40.4%,^23^ 39.5%,^21^ and 34.8%. Other studies claimed that defenders were the most vulnerable players 45%.^20^ According to Zarei et al., strikers accounted for the highest prevalence of injury (31%).^20^ Anderson et al. found that strikers and midfielders suffered more when attacking, and defenders and goalkeepers suffered more when defending.^24^ The injury rate, injury mechanisms, and injury locations appear to differ across various positions, indicating a need for additional research. There is no agreement on the impact of BMI on injury rates. Some research has proposed a negative correlation between BMI and injury occurrence, indicating that a higher body mass index is linked to a higher likelihood of lower limb injuries,^25-27^ and others have found no association between the effect of BMI on the incidence of injury.^28,29^

In most previous studies, video analysis has been used for data collection.^20,24^ Therefore, it is important to conduct a study using a direct examination method by a physician based on the standard protocol of PCMA for soccer players.

Based on recent epidemiological research, it is necessary to constantly update information about the prevalence of injuries and to investigate the role of variables related to their prevalence in any sport. Also, given the important role of study, recognition, and acquisition of information on the prevention program in any sports injury, this study aimed to examine the prevalence of sports injuries and their correlation with body mass index and player position on the field among Iranian soccer players.

## Methods 

This cross-sectional study was conducted for one month. on professional soccer players of the Iranian Soccer Pro League who were referred to the Iranian Center for Medical Assessment and Soccer Rehabilitation at the national soccer team camp to participate in pre-season medical examinations.

**Eligibility criteria**

Inclusion criteria: 1) professional soccer players of the Iranian Soccer; 2) man having at least three years of football experience

Exclusion criteria: 1) Unwillingness to participate in the study 

All participants provided written consent and agreed to participate in the study. The study received ethical approval from the Medical Ethics Committee of Hamadan University of Medical Sciences (IR.UMSHA.REC.1397.748).

In this study, the census method was used to collect data. We used a personal data form to collect the individuals’ history and characteristics. Additionally, to measure the subjects’ height and weight, a gauge that was graded in centimeters and a digital scale (made by Novin Company in Iran) were used. To collect data, we used a standard pre-tournament medical evaluation protocol called PCMA, approved by the FIFA Medical Committee. Data on this form include age, type of current sports injury (contraction, contusion, strain, sprain, bruise, ligament injury, chin injury, fracture, and dislocation), location of current sports injury (shoulder, arm, elbow, forearm, wrist, hand, pelvis, thigh, knees, leg, foot, ankles, and spine), history of severe sports injury (one leading to an absence period more than four weeks from competitions), the player’s professional post in the field (striker, midfielder, defender, and goalkeeper), and the player’s BMI. This was recorded by a physician after the interview, examination, and evaluation of the soccer players.

SPSS software (version 21) was used for data analysis. Student t-test was used to compare demographic characteristics injured and healthy athlete. Chi square and fisher exact tests were used to assess the association between player position, injury type and BMI classification and relationship between sports injuries and the male national Soccer players’ post. The significant level was considered less than 0.05.

## Results

A total of 56 professional soccer players with an average age of 19.8 ± 1.3 years and a BMI of 20.5 ± 2.5 kg/m2 participated in this study. Among them, 15 players reported injuries and pain in one or more areas of the body. Table 1 presents the demographic characteristics of the injured and healthy athletes.

**Table 1:** Comparison of demographic characteristics and sports history between injured and healthy soccer players

Demographic characteristics	Injured Athletes	Healthy Athletes	P- Value*
**Age**	19.77 ±1.32	20.25±1.16	0.340
**Weight**	69.27±7.60	74±6.71	0.104
**Height**	179.41±7.77	179.21±9.52	0.947
**Body Mass Index(kg/m2)**	21.66±3. 27	23.16±2.88	0.227
**Sports History**	24.16±14.08	23±14.02	0.829

The results of the independent samples t-test revealed no statistically significant differences in the demographic characteristics between the injured and healthy athletes (p > 0.05). The mean age was 19.77 ± 1.32 years for injured athletes and 20.25 ± 1.16 years for healthy athletes (p = 0.340). The mean weight was 69.27 ± 7.60 kg for injured athletes and 74 ± 6.71 kg for healthy athletes (p = 0.104). The mean height was 179.41 ± 7.77 cm for injured athletes and 179.21 ± 9.52 cm for healthy athletes (p = 0.947). The mean BMI was 21.66 ± 3.27 kg/m2 for injured athletes and 23.16 ± 2.88 kg/m2 for healthy athletes (p = 0.227). The mean sports history was 24.16 ± 14.08 months for injured athletes and 23 ± 14.02 months for healthy athletes (p = 0.829). 

Table 2 presents the incidence rates and relationships between various types of sports injuries and BMI categories among 56 male national soccer players. The data indicates that a total of 68 injuries were reported, with the most common being knee injuries (48.21%), followed by knee pain (21.43%), groin injuries (12.5%), hamstring injuries (8.93%), and ankle injuries (30.36%). The BMI categories were divided into underweight (17-18.5), normal (18.5-25), and overweight (25-30). Among the injuries, the majority of hamstring injuries (80%) occurred in players with a normal BMI, while 28.57% of groin injuries were seen in underweight players. Knee pain was predominantly reported in normal BMI players (83.33%), and knee injuries were most prevalent among normal BMI players (66.67%). Ankle injuries were reported in 70.59% of normal BMI players. The p-values for the relationship between injury types and BMI categories ranged from 0.36 to 0.99, indicating no statistically significant associations. Overall, these findings suggest that while certain injury types are more prevalent in specific BMI categories, the relationships observed were not statistically significant.

**Table 2:** Relationships between various types of sports injuries and BMI categories among male national soccer players.

Types of sports injuries	Total (%)	BMI⃰⃰⃰			P- Value*
		Under weight(17 - 18.5)	Normal (18.5-25)	Over Wight (25 - 30)	
**Hamstring injury**	5 (8.93)	0 (00.00)	4 (80.00)	1 (20.00)	0.67
**Groin**	7 (12.5)	2 (28.57)	4 (57.14)	1 (14.29)	0.36
**Knee pain**	12 (21.43)	1 (8.33)	10 (83.33)	1 (8.33)	0.50
**Knee injury**	27 (48.21)	3 (11.11)	18 (66.67)	6 (22.22)	0.70
**Ankle injury**	17 (30.36)	2 (11.76)	12 (70.59)	3 (17.65)	0.99
**Total**	68	8	48	12	

*Fisher exact test

The results of the Pearson test also revealed no significant correlation between sports injuries and BMI (p <0.05).

The findings presented in Table 3 outline the incidence of various sports injuries among players in different positions on the field, including strikers, goalkeepers, halfbacks, and defenders. For knee pain, a significant association was observed, with 50% in halfbacks, compared to 33.33% in strikers and 16.67% in goalkeepers, while no defenders reported knee pain (p = 0.04). In contrast, groin injuries did not show a significant relationship across positions, with 57.14% in strikers (p = 0.49). Hamstring injuries were similarly distributed, with no significant differences noted among positions, as 40% in halfbacks (p = 0.79). Knee injuries were reported 33.33% in strikers and 29.63% in defenders, but again, no significant association was found (p = 0.94). Lastly, ankle injuries were reported 29.41% in strikers and halfbacks, with no significant differences across positions (p = 0.93). 

**Table 3:** The relationship between sports injuries and the male national Soccer players’ post in the field (N₌56).

Types of sports injuries	Injury	players’ post in the field				P- Value
		Striker N=17	Goalkeeper N=9	Halfback N=14	Defender N=16	
**Knee pain**	Yes	4(33.33)	2 (16.67)	6 (50.00)	0 (0.00)	0.04*
	No	13 (29.55)	7 (15.91)	8 (18.18)	16 (36.36)	
**Groin**	Yes	4 (57.14)	1 (14.29)	1 (14.29)	1 (14.29)	0.49*
	No	13 (26.53)	8 (16.33)	13 (26.53)	15 (30.61)	
**Hamstring injury**	Yes	1 (20)	1 (20)	2 (40)	1 (20)	0.79*
	No	16 (32)	8 (16)	11 (22)	15 (30)	
**Knee injury**	Yes	9 (33.33)	4 (14.81)	6 (22.22)	8 (29.63)	0.94**
	No	8 (27.59)	5 (17.24)	8 (27.59)	8 (27.59)	
**Ankle injury**	Yes	5 (29.41)	3 (17.65)	5 (29.41)	4 (23.53)	0.93*
	No	12 (30.77)	6 (15.38)	9 (23.08)	12 (30.77)	

*Fisher exact test, ** chi square test

Table 4 presents the distribution of players' BMI categories across different positions on the field, including strikers, goalkeepers, halfbacks, and defenders. Among strikers, the majority fell within the normal BMI category, with 77.77% classified as normal weight, while only 5.56% were underweight and 16.67% were overweight. In the goalkeeper position, 44.44% were classified as overweight, with 33.33% underweight and 22.22% normal weight. Halfbacks also showed a predominance of normal weight players at 64.29%, with 11.11% underweight and 21.43% overweight. Defenders had a similar distribution, with 77.78% in the normal BMI range, 11.11% underweight, and 11.11% overweight (P=0.111). 

**Table 4:** Relationship between body mass index and player position (N₌56).

players’ post in the field	BMI			P- Value*
	Under weight	Normal	Over Wight	
**Striker**	1 (5.56)	13 (77.77)	3 (16.67)	0.111
**Goalkeeper**	3 (33.33)	2 (22.22)	4 (44.44)	
**Halfback**	2 (11.11)	9 (64.29)	3 (21.43)	
**Defender**	2 (11.11)	12 (77.78)	2 (11.11)	

*Fisher exact test

## Discussion

The present research demonstrated that 24.16±14.08 of the male national players suffered from an injury at the time of pre-competitions and pre-competitions-competition assessment. Knee injury 27 (48.21), ankle injury 17 (30.36), knee pain 12 (21.43), groin pain 7 (12.5), and hamstring injury 5 (8.93), were the most common injuries, respectively.

This finding, which is in agreement with several studies, was expected considering the involvement of most of the lower extremities in soccer. Angorani et al. noticed that the majority of injuries were associated with lower extremities, especially knee ligament injury (42%).^15^ Studies have also indicated that knee, thigh, ankle, tackling, being tackled, and collision mechanisms are the most important causes of injuries.^30^ In another study conducted on 390 Iranian professional soccer players from 16 clubs, Rahnama et al. noted that knee injury had the highest rate, and anterior cruciate ligament injury was the most vulnerable part of the knee area to injury.^16^ Forsythe's study revealed that the most frequent injuries among professional soccer players in the United States were hamstring strains (12.3%) and ankle sprains (8.5%).^31^ The ankle is particularly susceptible to injury during running, passing, and kicking the ball. These actions, which involve striking the ball with the inside of the foot and forefoot, can place the ankle in a compromised position, leading to external rotation. 

One of the contradictory studies includes the study conducted by Hassabi et al. They aimed to investigate the incidence of injuries in PAS soccer players, lower limb injuries, among which, muscle spasms and contusions were regarded as the most common type of injury among professional soccer players in Iran.^32^ Goodrich, Ezra, et al. identified the most common injury as an ankle sprain.^33^ Zarei et al. identified the leg as the body part with the highest injury incidence (20%), which could be due to different training programs as well as differences in the quality of the playing field and the training of different teams.^20^ Furthermore, the method of injury assessment is different in different studies. For example, Angorani et al. used the standard protocol of pre-competition medical evaluation called PCMA.

The most common causes of knee pain and knee injuries are common knee injury mechanisms, such as tilting, tackling, running with sudden acceleration, rotation,^34,35^ and valgus collapse of the knee, which are the most important mechanisms of injuries, including anterior cruciate ligament rupture in soccer.^36^ It appears that striker are more subject to these conditions owing to their role, performance, and presence in the penalty area.

The results of this study on player position and its effect on the rate of injury revealed that knee injury, knee pain, groin pain, and ankle injury were more common among strikers. The only significant relationship was between knee pain and the attacking position, and the striker had significantly more knee pain than the other positions.

Zaree et al., who studied injuries among Asian Cup soccer players, declared that the most prevalent injuries could be found in the lower body and occur in the goal area. They also stated that injuries in Halfback were prevalent to the extent of 50.0%, which was significantly higher than other positions.^36^ mcMaster and Walter found that attackers were the most vulnerable players to injury.^10^ In addition, according to one study by Arliani et al., which examined the two main rounds of professional soccer competitions in Sao Paulo, Brazil in 2019, the most common injuries occurred in attackers, and muscle injuries were the most common type of injury.^37^ Contradictory studies in this regard include the study of Angorani et al. who regarded the midfield position as the highest recurrence of injury (42%).^38^ Furthermore, in the study conducted by Svensson et al. (2018) on the elite male soccer players of Sweden, the highest prevalence of muscle injuries was reported in the lower torso and the defensive position (45%).^39^

One of the reasons for more injuries among striker can be their more activity and effort in scoring goals during the game and performing explosive movements and quick starts with high repetition.^40^ Furthermore, the tightness and frequency of the players in the penalty area and the penalty area provide more physical collision conditions. Some studies have mentioned the occurrence of more injuries near the post and the penalty area due to confrontations between striker and defenders.^24,41^

One of the reasons for the discrepancy in the results of such studies is a method through which data as well as history of related injuries are collected, which could make the comparison between the results difficult. For example, in a study aiming at specifying the prevalence of sports injuries using video analysis, any injuries leading a player to need medical team and treatment at the time of competition could be regarded as an injury, while in the current work, we only considered those pains and injuries reported by players referring to the Iran Soccer Medical Assessment and Rehabilitation Center before competitions.

In this study, no significant relationship was observed between BMI and injury rate. Turbeville's study shows The physical characteristics of players, such as BMI and strength, were not associated with risk of injury^42^ but Gashi F et al.^43^ found that greater BMI was associated with lower-extremity injuries in elite female soccer players.

Although hamstring, knee, and ankle injuries were higher in players with normal BMI, they were not statistically significant according to the sample size. It appears that the low number of samples in other categories of BMI (lean and obese) has been the reason for not achieving a significant relationship. The number of samples in the lean group was one, and in the obese group, there were no samples. 

In Angorani et al., there was no significant relationship between BMI and the rate of injury among soccer players.^15^

Additionally, given the limitation of BMI to differentiate between two masses of muscle and fat, there is the possibility of individuals with greater muscle mass being ranked in the overweight group, which could affect the validity of the results. However, the association between muscle mass and the occurrence of sports injuries in soccer players is unclear and needs further investigation. Therefore, the use of methods in determining the body type of soccer players in the PCMA protocol that directly measures their body fat percentage and muscle mass is recommended. More studies are needed in this field due to the lack of studies that have dealt with this relationship and the inconsistency in the results of previous studies regarding the effect of body mass index on injuries among soccer players. The limitation of this study was that it was carried out cross-sectionally during the examinations before the Iranian football season, and naturally, the prevalence of sports injuries in Iranian professional football is significant for investigating the risk factors of injury in football. 

## Conclusion

Given that treatment of sports injuries like knee ligament injuries, particularly the anterior cruciate ligament, needs surgery and long absence from competitions, which could impose heavy costs on the Pro League of the country, taking precautionary measures to prevent sports injuries is highly important. Therefore, it is recommended that further comprehensive studies be conducted to explore the factors influencing the occurrence of sports injuries in soccer players. 

It can be concluded that a high prevalence of injuries in the knee joint of soccer players was observed and there is a significant relationship between pain in the knee area and the position of the player on the field. Therefore, knee pain in attacking players was significantly higher than in other positions on the field.

Therefore, it is recommended that athletes, coaches, and all those involved in the team consider the above-mentioned issues in explaining sports injury prevention programs in soccer.

**Acknowledgment**

The author would like to thank the Clinical Research Development Unit (CRDU) of Besat Hospital, Hamadan University of Medical Sciences, Hamadan, Iran for their support, cooperation and assistance throughout the period of study. (Grant number 9710186166)" 

**Author Contribution:**

TSB: Study design, interpretation of the results and drafting the manuscript; SKH: Interpretation of the results, and drafting the manuscript; HN: Interpretation of the results and drafting the manuscript; BH: Interpretation of the results and drafting the manuscript; SKH: Statistical analysis, HN: Study design, acquisition of data, interpretation of the results.
